# European evidence-based recommendations for clinical assessment of upper limb in neurorehabilitation (CAULIN): data synthesis from systematic reviews, clinical practice guidelines and expert consensus

**DOI:** 10.1186/s12984-021-00951-y

**Published:** 2021-11-08

**Authors:** Gerdienke B. Prange-Lasonder, Margit Alt Murphy, Ilse Lamers, Ann-Marie Hughes, Jaap H. Buurke, Peter Feys, Thierry Keller, Verena Klamroth-Marganska, Ina M. Tarkka, Annick Timmermans, Jane H. Burridge

**Affiliations:** 1 Roessingh Research and Development, Enschede, The Netherlands; 2 Department of Biomechanical Engineering, University of Twente, Enschede, The Netherlands; 3 Institute of Neuroscience and Physiology, University of Gothenburg, Gothenburg, Sweden; 4 Rehabilitation Research Center (REVAL), UHasselt, Diepenbeek, Belgium; 5 Rehabilitation and MS Center, Pelt, Belgium; 6 School of Health Sciences, University of Southampton, Southampton, UK; 7 Department of Biosignals and Systems, University of Twente, Enschede, The Netherlands; 8 Neurorehabilitation Area at the Health Division of TECNALIA, Basque Research and Technology Alliance (BRTA), Donostia - San Sebastian, Spain; 9 Institute of Occupational Therapy, Zurich University of Applied Sciences, Winterthur, Switzerland; 10 Faculty of Sport and Health Sciences, University of Jyväskylä, Jyväskylä, Finland

**Keywords:** Upper limb, Upper extremity, Assessment, Rehabilitation, Therapy, Outcome measures, Stroke, Traumatic brain injury, Spinal cord injury, Multiple sclerosis

## Abstract

**Background:**

Technology-supported rehabilitation can help alleviate the increasing need for cost-effective rehabilitation of neurological conditions, but use in clinical practice remains limited. Agreement on a core set of reliable, valid and accessible outcome measures to assess rehabilitation outcomes is needed to generate strong evidence about effectiveness of rehabilitation approaches, including technologies. This paper collates and synthesizes a core set from multiple sources; combining existing evidence, clinical practice guidelines and expert consensus into European recommendations for Clinical Assessment of Upper Limb In Neurorehabilitation (CAULIN).

**Methods:**

Data from systematic reviews, clinical practice guidelines and expert consensus (Delphi methodology) were systematically extracted and synthesized using strength of evidence rating criteria, in addition to recommendations on assessment procedures. Three sets were defined: a *core set*: strong evidence for validity, reliability, responsiveness and clinical utility AND recommended by at least two sources; an *extended set*: strong evidence OR recommended by at least two sources and a *supplementary set*: some evidence OR recommended by at least one of the sources.

**Results:**

In total, 12 measures (with primary focus on stroke) were included, encompassing body function and activity level of the International Classification of Functioning and Health. The *core set* recommended for clinical practice and research: Fugl-Meyer Assessment of Upper Extremity (FMA-UE) and Action Research Arm Test (ARAT); the *extended set* recommended for clinical practice and/or clinical research: kinematic measures, Box and Block Test (BBT), Chedoke Arm Hand Activity Inventory (CAHAI), Wolf Motor Function Test (WMFT), Nine Hole Peg Test (NHPT) and ABILHAND; the *supplementary set* recommended for research or specific occasions: Motricity Index (MI); Chedoke-McMaster Stroke Assessment (CMSA), Stroke Rehabilitation Assessment Movement (STREAM), Frenchay Arm Test (FAT), Motor Assessment Scale (MAS) and body-worn movement sensors. Assessments should be conducted at pre-defined regular intervals by trained personnel. Global measures should be applied within 24 h of hospital admission and upper limb specific measures within 1 week.

**Conclusions:**

The CAULIN recommendations for outcome measures and assessment procedures provide a clear, simple, evidence-based three-level structure for upper limb assessment in neurological rehabilitation. Widespread adoption and sustained use will improve quality of clinical practice and facilitate meta-analysis, critical for the advancement of technology-supported neurorehabilitation.

## Background

Neurological conditions are a leading cause of disability world-wide. Incidence is rising due to an ageing world population and prevalence is increasing due to growth of the world population, better survival rates and improved long-term care [1]. The result is increasing pressure on the healthcare system globally and frames the need for effective and efficient approaches to enable and maintain access to care.

Recent advances in neurorehabilitation research have resulted in a better understanding of recovery, giving rise to new promising approaches such as increased intensity of practice, early intervention and use of technology. Of those, the use of technology in rehabilitation may help alleviate the pressure on the healthcare system. Moreover, technologies could enable access to rehabilitation throughout the lifespan and has been advocated by the World Health Organisation (WHO) as an investment in human capital that contributes to health, economic and social development [2].

For a successful transfer of therapeutic interventions using rehabilitation technology into clinical practice, evidence of their effectiveness is essential. This is reflected in national strategies and frameworks emphasising the need for informed decision making in healthcare that is research-led and evidence-based. Yet, several national guidelines cite limited research evidence to justify the use of rehabilitation technologies [3–5]. Indeed, data on clinical evaluations of interventions in neurological rehabilitation, either conventional or technological, are not easily comparable due to inconsistency in what is actually measured [2], and the measurement tools used. Consequently, there is a paucity of high-quality evidence from systematic reviews and meta-analyses [6].

Agreement on outcome measures (OM) and corresponding procedures for assessment are critical to advancing the field. For new approaches to be used effectively in clinical practice (the right therapy approach with the right patients, at the right time and delivered via the most effective protocols), clinicians need clear assessment guidelines to enable them to make informed decisions. The use of agreed, uniform OM is not only useful in order to compare the effectiveness of different training approaches, but also to identify which patients benefit most from which training approach and dose.

For example, the use of different technologies for task-oriented training of the upper limb was investigated in highly functional chronic stroke patients in two separate clinical trials using a sensor system [7] or a robot system [8]. As both studies used the same OM, results could be combined, showing that training with the inertial sensor system providing feedback on exercise performance was more beneficial for highly functional patients than the robot-guided system [9].

In addition, practical and accurate tools are emerging that can predict recovery, with the potential to significantly improve patient management and reduce costs of health services [10]. Establishing and elaborating clinical prediction models for the upper limb, such as SAFE [11] and PREP2 [12], to facilitate personalisation of patient rehabilitation and discharge planning, can only occur if sufficient good quality objective assessment data is available.

The European Network on Robotics for Neurorehabilitation (European Co-operation in Science and Technology, COST Action TD1006) has developed a set of recommendations for upper limb assessment in neurological conditions, to evaluate both conventional and technology-supported therapy. These European recommendations aim to improve the quality of upper limb neurorehabilitation in clinical practice globally, through the adoption of standardised, agreed protocols for assessment in clinical practice and research. The recommendations will directly support clinical research and facilitate larger scale multi-centre studies, allowing meta-analyses, essential for informing and stimulating investigation of prediction for patient-specific training approaches and more generally advancing understanding of recovery. It will also inform and influence the development of new upper limb neurorehabilitation technologies both as therapies and assessment tools, and assist in the translation of useful technologies into clinical practice.

The present paper collates and synthesizes the recommendations from multiple sources, combining existing evidence, current clinical practice guidelines and expert consensus, into the recommendations for Clinical Assessment of Upper Limb In Neurorehabilitation (CAULIN). The CAULIN recommendations provide evidence-based recommendations for upper limb assessment of patients with neurological conditions before, during and after therapy (either conventional or technology-assisted treatment), including the recommended time frame of applying structured assessment where available.

## Methods

### Scope and purpose

The CAULIN recommendations were developed within the framework of a European network (COST Action TD1006). This enabled involvement of more than 200 experts and stakeholders from over 24 European countries, with a wide range of backgrounds: physical therapists, occupational therapists, physicians and nurses (all working primarily in neurorehabilitation); clinical researchers from the same or related professions; engineers, technology developers; neurological patients; and other stakeholders such as neurorehabilitation educators and healthcare insurers.

A systematic approach, in correspondence with the Appraisal of Guidelines for Research and Evaluation (AGREE) II methodology [13], addressing particularly the AGREE domains of scope and purpose, stakeholder involvement and rigour of development, was used. Both clinical and technology-generated outcome measures were considered, using an expanded version of the WHO International Classification of Functioning, Disability and Health (ICF) as the structuring model, distinguishing at the activity level between capacity (i.e., maximal ability measured in a controlled setting) and performance (i.e., level of functioning in a person’s current environment), with performance further divided between perceived (subjective experienced by a person) and actual (objectively measured) performance. OM on participation level are not targeted specifically for the CAULIN recommendations, considering that participation OM assess more complex activities and social life situations, which aren’t strongly related to UL functioning [14].

Although the evidence and information on which the recommendations are based focus primarily on stroke, other neurological conditions are addressed as well, including spinal cord injury (SCI), multiple sclerosis (MS), and traumatic brain injury (TBI). The recommendations are primarily targeted at supporting clinicians during clinical decision making, but they are also applicable to all professionals working in neurorehabilitation, including research, to establish uniform methods for reporting clinical outcomes.

### Procedure for development of recommendations

**Fig. 1:**
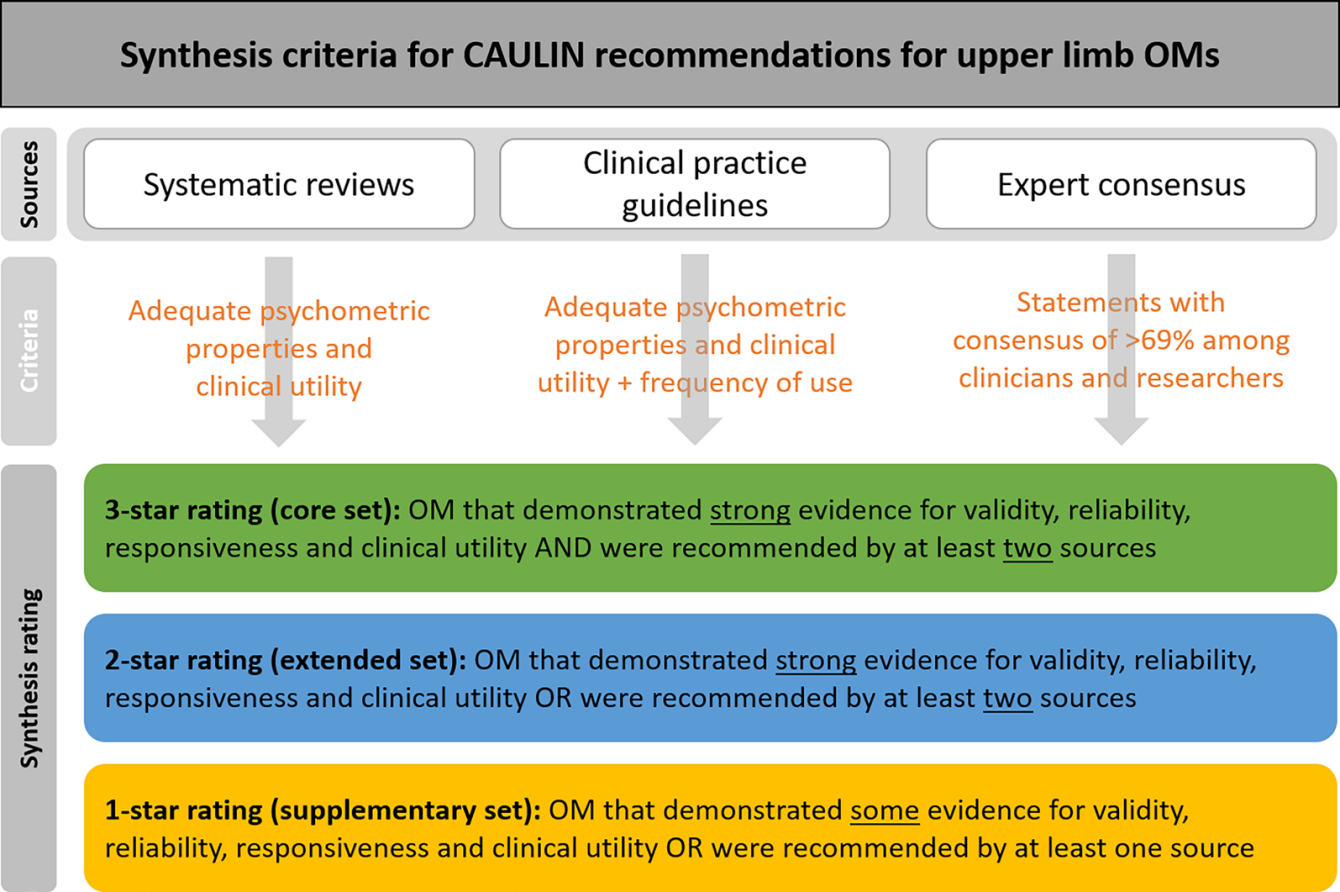
Schematic view of synthesis criteria for compiling CAULIN recommendations

A structured approach was applied to generate the recommendations, synthesizing three published sources of evidence: existing scientific literature, clinical practice guidelines and expert consensus (Fig. 1). Scientific evidence was provided by a systematic overview of systematic reviews on upper limb OM in stroke including evaluation of psychometric properties and clinical utility [15]. An extensive survey of existing clinical practice guidelines provided recommendations and clinical evidence on assessment and OM across different neurological conditions [16]. Agreed expert opinions on use of OM for assessment of the upper limb in neurorehabilitation were derived from a Delphi consensus study among the 24 European Union (EU) member countries of the COST Action, involving 60 clinicians, 35 clinical researchers, 77 non-clinical researchers and 35 engineers [17]. Each of these research activities were coordinated and executed by members of the TD1006 COST Action (Working Group 1). These activities took largely place in parallel. With this paper we integrate their outcomes.

The CAULIN recommendations for upper limb assessment in neurorehabilitation include:

1. A recommendation on specific sets of OM on body functions and structures and activity level,
2. A recommendation on assessment procedures specifying when, how and by whom the assessments should be done.

### Sources of information

#### Systematic reviews

The systematic overview of 13 systematic reviews (published between 2004 and 2014) focused on the psychometric properties and clinical utility of upper limb OM in stroke [15]. From 53 different upper limb OM included in the overview, 13 met the standards and criteria set for the validity, reliability, responsiveness and clinical utility. Of those, six OM demonstrated a high level of measurement quality and clinical utility and were recommended for assessment of upper limb function and activity in research and clinical practice. All 13 OM with published evidence of adequate measurement quality (psychometric properties) and clinical utility were considered for the synthesized CAULIN recommendations.

### Clinical practice guidelines

The evidence from 34 records (published between 2007 and 2017), including existing national clinical guidelines and published practice guidelines, on assessment of upper limb in neurorehabilitation provided input from clinical practice to the CAULIN recommendations [16]. The specific OM of body function, activity and participation recommended by these clinical practice guidelines for upper limb assessment were considered for the current synthesis.

### Expert consensus

A Delphi consensus exercise with six consensus rounds performed between 2011 and 2015 provided evidence from five expert groups, consisting of 208 clinicians and researchers from medical and engineering fields across Europe. In each expert group, votes were collected on questions and statements about the use of OM for upper limb assessment in neurorehabilitation. At least 69% consensus was required for each statement to be included as a recommendation for the current synthesis [17].

### Structured data synthesis

#### Recommended OM sets

Data from all three sources (systematic reviews, clinical practice guidelines, and expert consensus) were systematically extracted and combined to form specific sets of recommended OM (Fig. 1). The extracted data were synthesized across the three sources by rating them, based on the strength of evidence, according to the following criteria:

- *Core set* (3-star rating): OM that demonstrated strong evidence for validity, reliability, responsiveness and clinical utility AND were recommended by at least two sources.
- *Extended set* (2-star rating): OM that demonstrated strong evidence for validity, reliability, responsiveness and clinical utility OR were recommended by at least two sources.
- *Supplementary set* (1-star rating): OM that showed some evidence for validity, reliability, responsiveness and clinical utility OR were recommended by at least one of the sources.

The core (3-star) and extended (2-star) sets of CAULIN recommended OM represent OM that are psychometrically sound, have suitable clinical utility and have a solid support base in the clinical and research community. The core 3-star OM should, however, always be considered as a first choice for all clinical trials and implementation protocols in clinical settings. The 1-star rated OM represent those with good potential, but where the psychometric properties, clinical utility or expert consensus is not fully established. These measures could be used where appropriate or for research purposes. For example, additional specific OM might be needed when investigating specific treatments, such as robot-assisted therapy or home-based therapy, or when patients present with specific problems or treatment goals.

### Assessment procedures

Two of the three sources, i.e., clinical practice guidelines and expert consensus, generated data on recommended procedures for assessment of upper limb functioning. Based on available information, data was extracted and categorized according to three characteristics: time spent in assessment; frequency and timing of assessments; person who should conduct the assessments. Due to limited data available, rating of recommendations as done with OM selection couldn’t be applied to assessment procedures, instead data synthesis consisted of summarizing and categorizing the evidence.

## Results

### Recommended OM

**Fig. 2:**
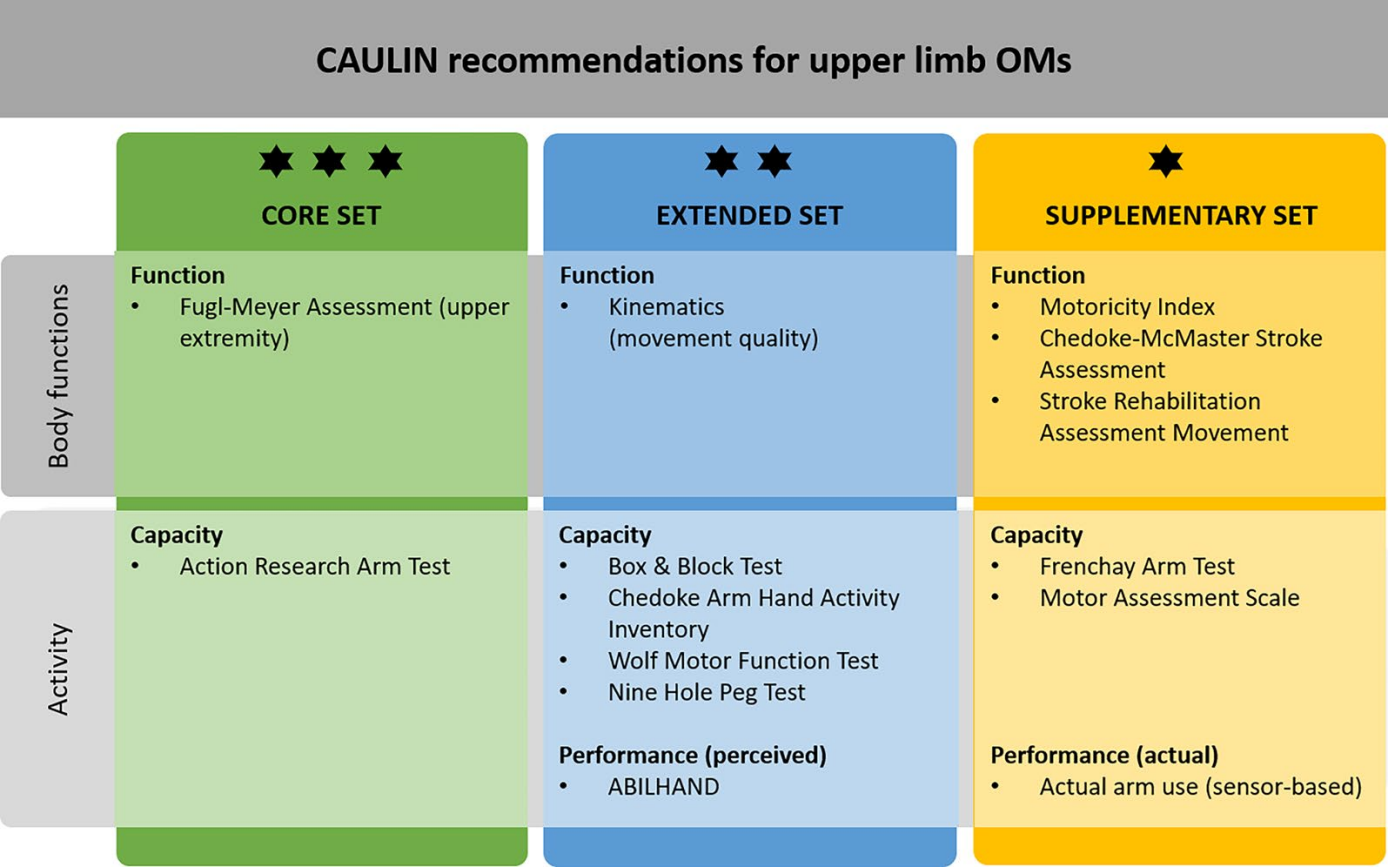
CAULIN recommendations for selected specific upper limb outcome measures in neurorehabilitation

The synthesized results for the CAULIN recommendations on specific OM are shown in Table 1. A general recommendation concerning the scope of upper limb assessment has been highlighted across the three data sources: OM must be valid, reliable, responsive, clinically available and useful, preferably a consolidated set. In total, 12 specific OM were included, covering body function and activity level of the ICF (Fig. 2).

**Table 1:** Overview of synthesized data from systematic review of OM, review of clinical practice guidelines and expert consensus

Systematic reviews	Practice guidelines	Expert consensus	CAULIN recommendation
OM for body functions			
Recommendation: Strong evidence for FMA-UE; Some evidence for MI-arm, CMSA, STREAM, kinematics	Recommendation: No specific set of OM was recommended, although the FMA-UE were most frequently recommended in the included stroke guidelines; Note: psychometric properties of many OM are not established (e.g. spasticity)	Recommendation: No consensus was reached on specific OM Recommended for clinicians and researchers: A defined core set including validated clinical OM but also less establish OM with potential for special circumstances and for research; technology-generated measures (e.g. kinematics and wearables) Recommended for use in research: Quality of movement execution, neurophysiological (EMG, TMS), neuroimaging Recommendations for clinicians: Effort and amount of assistance (e.g. in robotics)	FMA-UE is the most often recommended OM of UL impairment and has strong psychometric properties Some evidence to use kinematics (to measure movement quality) Some evidence to use MI-arm, CMSA, STREAM
OM for activity			
Recommendation: Strong evidence: ARAT, BBT, CAHAI, WMFT (activity capacity); Some evidence: FAT, MAS, NHPT; Generally recommended: Body-worn sensors to measure activity in daily life (limited evidence on psychometrics)	Recommendation: No specific set of OM was recommended, although ARAT, NHPT, FIM, BI are most frequently recommended in the stroke guidelines Generally recommended: Body-worn sensors to measure activity in daily life and monitor adherence to exercise programs	Recommendation: No consensus was reached on specific OM Recommended for clinicians and researchers: A defined core set of established validated clinical OM Recommended for researchers: Body-worn sensors to monitor activity performance	ARAT is the most often recommended OM of UL activity capacity and has strong psychometric properties BBT, CAHAI, WMFT have strong psychometric properties Some evidence to use FAT, MAS, NHPT (of which NHPT recommended twice) FIM and BI are most often recommended generic ADL instruments (out of scope for UL-specific OM) Measures of actual arm use (body-worn sensors) should be considered
PROM			
Recommendation: Strong evidence: ABILHAND	Recommendation: Patient–reported outcomes should be used along with objective OM	Recommendation: Self-reported measures should be used	Patient–reported outcomes should be used along with objective OM ABILHAND has strong psychometric properties
Goal-oriented OM			
Recommendation: Not applicable (goal-oriented instruments were not included)	Recommendation: Goal attainment OM to link assessment to goal setting and to motivate patients should be used along with objective OM	Recommendation: Personalized goal attainment measures should be used	Goal attainment OM should be used along with objective OM No specific OM can be recommended

FMA-UE, Fugl-Meyer Assessment, Upper Extremity part; MI-arm, Motricity Index, arm part; CMSA, Chedoke-McMaster Stroke Assessment; STREAM, Stroke Rehabilitation Assessment Movement; OM, outcome measure(s); EMG, electromyography; TMS, trans-cranial magnetic stimulation; ARAT, Action Research Arm Test; BBT, Box and Block Test; CAHAI, Chedoke Arm Hand Activity Inventory; WMFT, Wolf Motor Function Test; FAT, Frenchay Arm Test; MAS, Motor Assessment Scale; NHPT, Nine Hole Peg Test; FIM, Functional Independence Measure; BI, Barthel Index; UL, upper limb; PROM, patient-reported outcome measures

The recommended *core set* (3-star rating) of OM for clinical practice consists of Fugl-Meyer Assessment of Upper Extremity (FMA-UE) and Action Research Arm Test (ARAT). These were the only two measures presenting good psychometric properties while being recommended in at least two of the three data sources (systematic reviews of OM and clinical practice guidelines).

The *extended set* (2-star rating) adds six more OM recommended for clinical practice and/or clinical research. Kinematic measures assessing movement quality and execution are recommended at body function level, although there isn’t sufficient information available in the examined sources to specify which kinematic variable(s) should be used (i.e., range of motion, smoothness, etc.). Recommended OM to assess at activity level add four capacity measures with each a slightly different focus: Box and Block Test (BBT; timed unilateral gross motor dexterity), Chedoke Arm Hand Activity Inventory (CAHAI; focusing on bilateral task execution), Wolf Motor Function Test (WMFT; uni- and bilateral timed performance and ability scoring), Nine Hole Peg Test (NHPT, timed unilateral fine motor dexterity); and the ABILHAND (patient-reported manual ability measure).

The *supplementary set* (1-star rating) includes additional OM that can be used for specific research purposes. On body function level, the Motricity Index (MI), Chedoke-McMaster Stroke Assessment (CMSA) and Stroke Rehabilitation Assessment Movement (STREAM) are added. On activity level, the Frenchay Arm Test (FAT) and Motor Assessment Scale (MAS) are additional recommended OM to measure functional ability (activity capacity), as well as monitoring the amount of actual arm use in routine daily life (activity performance) through the use of body-worn movement sensors (e.g., accelerometers, inertial measurement units—IMU).

### Recommended assessment procedures

Although the extent of information available is limited on when and by whom assessments should be conducted, we have summarized the available evidence on assessment procedures from the three published data sources (Table 2), as follows:

1. Assessments should be conducted at regular intervals during rehabilitation at a minimum of four time points (early, 3-, 6- and 12-months after onset).
2. Global measures should be applied within 24 h of hospital admission and upper limb specific measures within 1 week.
3. During a rehabilitation program, assessment should be made at baseline (beginning of the program), interim (during the program), final (end of the program), and follow-up (a set period of time after completion of the program).
4. Patients should always be assessed prior to discharge or transfer in order to support appropriate follow-up.
5. OM should be administered separately from treatment, last no longer than three hours and be conducted by healthcare professionals who are trained to use them.

**Table 2:** Recommendations for assessment procedures

Procedure	Practice guidelines	Expert consensus
Duration, frequency and timing of assessments	Stroke: Screen for impairment, activity limitations, participation restrictions, and environmental factors to direct treatment on admission and on transfer from hospital to community Stroke: Assessment within 48 h including: function, safety, physical readiness, and ability to learn and participate in rehabilitation Stroke: Medical and global outcomes, impairment and activity early post stroke, at 3 months and ideally at 6 and 12-months’ post stroke Stroke: Early assessment and planning of discharge Spinal Cord Injury: Pain, motor and sensory dysfunction assessment should be carried out within 24–48 h of admission and prior to discharge Stroke: NIHSS performed by trained, certified assessors within the first 24 h, and consider re-assessing prior to discharge from acute care Stroke: Measure at predefined times to monitor recovery e.g., within one week of admission and discharge (or when transferring care) end of the 1st week, 3rd and 6th month post-stroke. Consider measures before each multidisciplinary meeting	Assessments should take no longer than three hours (92% agreement by clinicians) Four face-to-face patient assessments per treatment programme: beginning, during and end, and at a set period of time after the end of the programme Except for data collected automatically by technology (100% agreement for clinical practice) Assessment should take place separately from treatment (96% agreement by clinicians)
Person who should conduct assessments	Stroke: Clinicians should be trained in the use of measurement scales Stroke: Healthcare professionals who have appropriate skills and training Stroke: Assessment conducted by specialist staff Stroke: Recommends multi-disciplinary medical assessment Multiple Sclerosis: Assessment should be conducted by a “healthcare professional with appropriate expertise in rehabilitation and MS” Stroke: Standardized rater training needs to be developed Stroke: Multi-disciplinary team assessment should be undertaken to establish the patient’s rehabilitation needs and goals	

## Discussion

By combining existing evidence on OM from literature reviews, a systematic overview of national clinical practice guidelines across Europe and beyond, and expert consensus on a pan-European level, we compiled uniform and agreed evidence-based recommendations for Clinical Assessment of Upper Limb In Neurorehabilitation (CAULIN). As such, CAULIN provides evidence-based recommendations for upper limb assessment of patients with neurological conditions, primarily stroke, before, during and after therapy (either conventional or technology-assisted therapy), to be used primarily in clinical applications, but also in clinical research. Furthermore, CAULIN defines the recommended time frame of applying structured assessment at four specific instances (early, 3, 6 and 12 months after admission). The CAULIN recommendations defined OM at three levels: *core set* (including 2 OM)*, extended set* (adding 6 OM)*, and supplementary set* (extending by 6 OM).

The core set recommends FMA-UE and ARAT to be included as the core (3-star) assessments of upper limb function and activity capacity in clinical practice. This is in agreement with the consensus-based recommendations of the Stroke Recovery and Rehabilitation Roundtable (SRRR) [18] and the results of a recent consensus-based Delphi study [19]. The current outcome strengthens the recommendation of the use of FMA-UE and ARAT in routine clinical practice by collating evidence, not only from consensus-based methods, but also from systematic reviews on existing literature and clinical practice guidelines. The global coverage of SRRR consensus further supports the pan-European CAULIN core set recommendations for assessing upper limb function and capacity. Even though FMA-UE and ARAT assess upper limb functioning at different levels of the ICF framework and measure different constructs, strong correlations exist between both OM [20, 21]. It is recommended to apply both FMA-UE and ARAT whenever possible to cover both aspects of functioning. A choice for one over the other can however be based on the patient-specific treatment goals if needed (e.g., if administration time is limited).

The extended (2-star) set includes a mix of performance-based and patient-reported OM (PROM), addressing both capacity and perceived performance of the arm in daily life. These assessments can be used as complementary assessments, depending on patient-specific treatment goals or needs in clinical practice, or on specific research objectives in clinical research. For example, while the BBT and NHPT are easy to implement into clinical practice (i.e., they are short and quick to administer), they will provide summary information on task outcome. On the other hand, some of the other recommended OM are more comprehensive and will take more time and training, while adding valuable information on task execution and strategies used by the patient. The CAULIN recommendations, however, emphasize that the core OM (FMA-UE and ARAT) should be prioritized over the extended and supplementary OM sets.

In addition, the CAULIN extended (2-star) set recommends kinematic measures for assessment of movement quality on body function level. This extends the information gained through clinical assessments about task execution with more detailed information about its underlying aspects, for example movement smoothness [22]. This recommendation is primarily applicable for evaluation of specific, well-established tasks (e.g. reaching or pointing) implemented in clinical practice or clinical research. The use of kinematic measures has been encouraged to allow distinction between behavioural motor recovery and compensation [23, 24]. Furthermore, kinematic measures enable detection of more subtle and fine-grained changes and are thought to provide valuable information for individual treatment planning and evaluation [25]. Similar to CAULIN recommendations, the 1st SRRR initiative could not recommend specific kinematic measures for clinical research [23]. A more recent 2nd SRRR initiative, however, did specify a set of consensus-based kinematic measures for clinical research trials [6]. These guidelines propose kinematic data to be collected during two standardized movement tasks: a reaching task in the horizontal plane and a functional 3D reach-to-grasp task, such as drinking from a glass [6].

The use of PROM is recommended in each of the three CAULIN sources for perceived activity performance, with the ABILHAND as the only specific tool demonstrating sufficiently strong psychometric properties and clinical utility. In line with the overall aim of rehabilitation, perceived performance measures add valuable and necessary information about a person’s experienced limitations of upper limb use in daily life [26]. In the present synthesis, PROM were mentioned in all sources to deserve attention during UL assessment, but specific PROM’s besides ABILHAND couldn’t be extracted. Other OM exist that might be suitable (more detailed information is in the publications of the three data sources), but more effort to establish specific PROM guidelines is needed.

The evidence-base for assessments included in the supplementary (1-star) set is smaller compared to recommended 3- and 2-star OM. The additional OM in the supplementary set will primarily be applicable for clinical research or in specific contexts of clinical practice, depending on research questions or patient-specific treatment goals, as each of these assessments have their particular focus and advantages. One of the OM included in the supplementary set is sensor-based assessment of actual arm use in daily life. This adds the perspective of actual performance in ecologically valid real-life settings to that of capacity measures, assessing the maximum score in a controlled setting, which are known to be incongruent [27]. Although a standardized way to implement sensor-based assessment of actual arm use as an activity performance measure couldn’t be established in the current work, studies have indicated that assessing the actual use of the affected arm in daily life with respect to the unaffected arm (using activity counts) provides insight in non-use of the affected arm and relates more to real-world arm use than functional outcome measures [28, 29]. Nevertheless, establishing the optimal way for application and analysis requires further research.

The use of goal attainment OM were mentioned in all three CAULIN sources, although no specific OM could be identified. Remarkably, a Cochrane review reported that only 3 out of 39 studies, investigating the effect of goal-setting on psychosocial outcomes during rehabilitation of people with acquired disability, used a goal attainment evaluation [30]. Goal Attainment Scaling was the only OM used in those studies. A clinical guideline on integrating goal setting into rehabilitation to inform individual treatment planning listed nine useful goal attainment OM, but also wasn’t able to suggest a specific goal attainment OM [31]. Nevertheless, the current findings underline that goal attainment OM should be considered in clinical practice and further research is needed to define suitable goal attainment OM.

In terms of assessment procedures, the current synthesis derived the following recommendation: administration of upper limb specific OM should be done by trained healthcare professionals within 1 week of admission to rehabilitation and repeated prior to discharge or transfer, with specific time points during rehabilitation (upon start, during, end of programme, with a follow-up assessment). Despite the recognized importance of structured administration procedures, more specific recommendations couldn’t be derived from the current synthesis. When considering only consensus-based evidence, specific advice on time points for clinical assessment has recently been proposed, with maximal 7 assessments across 12 months: within 3 days (OM at body functions level only), at day 7, at weeks 2, 4, 12, at 6 months, followed by every 6th month [19]. These proposed timepoints are generally in alignment with the procedures recommended in CAULIN. Beyond this, more explicit recommendations on administration procedures beyond timing of assessment are desired for better comparability of outcomes.

## Considerations and limitations

The current work has identified uniform and agreed OM for clinical assessment of upper limb with pan-European coverage, integrating evidence on psychometric properties with clinical practice guidelines and with evidence-based consensus among clinicians, researchers and engineers, considering also clinical utility in aspects such as language availability, affordability and practical applicability. Despite these strengths, several limitations and considerations should be noted.

Kinematic measures and sensor-based actual arm use are included in the current recommendations to quantify movement quality and arm/hand use on body function and activity level, even though clinical applicability isn’t well-established yet. Such technology-supported assessments are increasingly used in research [32, 33], but they haven’t found their way to large scale application in clinical practice. Current limitations are a lack of a standardized way to apply and analyse the data and missing information regarding its psychometric properties for the various scenarios [15, 34]. In case of kinematic assessment an additional, contemporary, limitation is the need for high-resolution, three-dimensional optoelectronic systems [6], limiting application to specialized clinical centres that have access to such advanced systems and expertise required for the corresponding analysis. Apparently, the expected added value of objective measurement of upper limb function or actual use is compelling enough to have caught the attention of both researchers and healthcare professionals [17]. This is based on the rapid ongoing technological developments of equipment suitable for use outside of expert labs, such as accelerometers, inertial measurement units (IMU’s) or markerless video-based systems. Although currently regarded as not mature or user-friendly enough for routine use in clinical practice [6], it is expected that this will become possible in the coming years. This will then enable measurement of kinematic data and/or actual arm use in clinical settings during therapy, on the ward and even at home, without the need for advanced optoelectronic systems. Based on the current synthesis, it is clear that this topic warrants further research.

Potential cultural differences that can influence the validity of task-based assessments (e.g., using cutlery) haven’t been directly addressed, even though language availability of OM has been considered. For example, for the FMA-UE official transcultural adaptations and validations are available [35–38]. Also, some of the clinical assessments that are part of the CAULIN recommendations are available in revised or shortened forms, optimising administration time or psychometric properties [39–41], but this hasn’t been taken into consideration in this work.

Furthermore, the current synthesis shows that the majority of information about upper limb assessment deals with stroke. Nevertheless, wherever available, the CAULIN recommendations have used information on upper limb assessment from other populations. Based on available clinical practice guidelines, most information besides stroke was available from TBI, followed by SCI and MS [16]. For MS, the OM in CAULIN recommendations are in alignment with a previous review recommending amongst others NHPT, BBT, ARAT and WMFT as appropriate OM for MS [42]. Likewise, in SCI the ARAT has been used and recommended as primary upper limb functional outcome measure in clinical trials [43, 44]. Therefore, although being represented to a smaller extent than the stroke population, available evidence endorses the applicability of OM in the CAULIN recommendations for other neurological populations (TBI, SCI, MS), while considering the suitability of specific OM for the target population (e.g., FMA-UE would be applicable to TBI but not to SCI, ARAT is developed for stroke but is also used in SCI and MS).

The current work showed that PROM, goal attainment OM and sensor-based assessment of actual arm use in daily life are important concepts to include in upper limb assessment, although concrete recommendations based on consensus, clinical utility and psychometric properties, can’t be provided at this point. More research is needed to establish specific measures and/or methods. In addition, technological development is required to mature measurement systems and methods for use in clinical practice or research. This is also valid for kinematic measures of movement quality, even though a basic application could be specified in the extended set of CAULIN recommendations. Increased availability of assessment of movement and task performance on ratio-level, considering such developments in the (near) future, enables better detection of underlying, detailed changes. This will add valuable information for prognosis of recovery and corresponding treatment planning on individual level, which can benefit the rehabilitation process [45].

It is, however, conceivable that any new or additional OM will meet the selection criteria as defined for the CAULIN recommendations at some point. Moreover, some of those new or additional measures could potentially outperform some of the OM in the current selection, especially those with subjective (by the tester) and ordinal-level scoring involved. This entails that the recommendations should be updated in the future on a regular basis, to incorporate additional measures and revisit the selection of recommended OM, when these become available. Nevertheless, this means that the current CAULIN recommendations are limited to OM currently available in clinical practice.

## Conclusions

The CAULIN recommendations for OM and assessment procedures provide a clear, simple, evidence-based three-level structure for upper limb assessment in neurological rehabilitation. OM in all three levels have proven psychometric properties as well as evidence derived from systematic reviews and expert consensus. The three levels are: (1) Core set: OM that should be applied routinely in clinical practice with neurological patients undergoing conventional or technology-enhanced upper limb rehabilitation; (2) Extended set: OM that may be useful in clinical practice but are recommended as standard for research, and (3) Supplementary set: OM for specific research purposes. The CAULIN recommendations provide a comprehensive framework, in the context of currently available OM, within which to investigate the effectiveness of (technology-supported) interventions and better understand which patients benefit from which training approach. This will facilitate treatment planning in clinical practice on patient-specific basis. Widespread adoption and sustained use of the recommendations will increase opportunities for data pooling and meta-analysis, critical for the advancement of neurological rehabilitation.

## Data Availability

Data sharing is not applicable to this article as no datasets were generated or analysed during the current study. Data that was used as input for the current synthesis have been published earlier [15–17].

## Abbreviations

CAULIN: Clinical Assessment of Upper Limb In Neurorehabilitation
WHO: World Health Organisation
OM: Outcome Measures
COST: European Co-operation in Science and Technology
AGREE: Appraisal of Guidelines for Research and Evaluation
ICF: International Classification of Functioning, Disability and Health
SCI: Spinal cord injury
MS: Multiple sclerosis
TBI: Traumatic brain injury
CP: Cerebral palsy
PD: Parkinson’s disease
EU: European Union
FMA-UE: Fugl-Meyer Assessment of Upper Extremity
ARAT: Action Research Arm Test
BBT: Box and Block Test
CAHAI: Chedoke Arm Hand Activity Inventory
WMFT: Wolf Motor Function Test
NHPT: Nine Hole Peg Test
ABILHAND: Patient-reported manual ability measure
MI: Motricity Index
CMSA: Chedoke-McMaster Stroke Assessment
STREAM: Stroke Rehabilitation Assessment Movement
FAT: Frenchay Arm Test
MAS: Motor Assessment Scale
IMU: Inertial Measurement Units
EMG: Electromyography
TMS: Trans-cranial Magnetic Stimulation
FIM: Functional Independence Measure
BI: Barthel Index
SRRR: Stroke Recovery and Rehabilitation Roundtable
PROM: Patient-Reported Outcome Measures
